# Ambient air quality and subjective stress level using Community Health Survey data in Korea

**DOI:** 10.4178/epih.e2018028

**Published:** 2018-06-28

**Authors:** Myung-Jae Hwang, Hae-Kwan Cheong, Jong-Hun Kim, Youn Seo Koo, Hui-Young Yun

**Affiliations:** 1 Department of Social and Preventive Medicine, Sungkyunkwan University School of Medicine, Suwon, Korea; 2 Department of Environmental and Energy Engineering, Anyang University, Anyang, Korea

**Keywords:** Air pollution, Air quality, Stress, Quality of life, Community Health Survey, Korea

## Abstract

**OBJECTIVES:**

Air pollution causes various disease in exposed populations, and can lead to premorbid health effects manifested as both physical and psychological functional impairment. The present study investigated the subjective stress level in daily life in relation to the level of air pollution.

**METHODS:**

Data from the Community Health Survey (2013), comprising 99,162 men, and 121,273 women residing in 253 healthcare administrative districts, were combined with air pollutant concentration modelling data from the Korean Air Quality Forecasting System, and were stratified by subjective stress levels into five strata for multiple logistic regression. Levels of exposure were divided into five quintiles according to the annual concentration of nitrogen dioxide (NO_2_), and were analyzed using a single-pollutant model using NO_2_ concentration only, and a multi-pollutant model adjusted for the concentration of particulate matter <10 μm in diameter.

**RESULTS:**

Analysis of men and women in various age groups showed the highest odds ratio (OR) for subjective stress level at the highest NO_2_ concentration quintile in men and women aged 30–64 years (men: 2.91; 95% confidence interval [CI], 2.12 to 4.01; women: 1.82; 95% CI, 1.32 to 2.51). As the NO_2_ concentration quintile increased, the OR increased. Men showed higher ORs than women in all strata.

**CONCLUSIONS:**

In the present study, annual NO_2_ concentrations were found to be associated with subjective stress levels. This association was especially clear among socioeconomically active men and women aged 30-64 years.

## INTRODUCTION

Rapid industrialization and urbanization have led to severe air pollution, and subsequently, health problems worldwide. Fossil fuels, which are used in energy industries and vehicles, are a major cause of air pollution that leads to health problems. They release air pollutants such as particular matter (PM), nitrogen dioxide (NO_2_), carbon monoxide, and ozone. Inhaled air pollutants cause oxidative damage in lung cells, which induces lung diseases and bronchitis. Continued damage due to long-term exposure can lead to lung cancer. Inhaled particles enter the bloodstream, and induce a systemic inflammatory response, affecting blood vessels, and coagulation, and acutely affecting the autonomic nervous system [1]. Air pollutants not only worsen cardiovascular diseases and metabolic disorders, such as diabetes and hypertension, but also affect neurodegenerative diseases such as depression, autism spectrum disorder, Alzheimer’s disease and Parkinson’s disease [2- 5]. In 2012, the World Health Organization estimated the global number of deaths due to air pollution including indoor and ambient air pollution to be 7 million per year, which corresponds to 12.5% of all deaths [6]. Air pollution has been recognized as a major risk factor in public health. It has consistently caused problems in public health in not only developing countries, but also developed countries. Research on deaths or diseases due to exposure to air pollution has been actively conducted both domestic and international scale.

However, most studies have focused on deaths, disease incidence and disease morbidity due to air pollution. When exposed to air pollution, not just major health effects including disease, but physical and psychological functional loss can occur even before disease onset. These effects can be reflected by the emotions experienced in one’s daily life, such as discomfort, stress, annoyance, and anger, and even affect health-related quality of life (QoL). In 2000, the Swiss Study on Air Pollution and Lung Diseases in Adults found a strong correlation between concentration of PM < 10 μm in diameter (PM_10_) and NO_2_, and annoyance, in which the level of exposure increased in proportion to the subjects’ annoyance scores (r> 0.85) [7]. Therefore, exposure to air pollution can affect the subjective emotional states of the individual. Epidemiologically, the association of air pollution exposure with subjective and objective scales of emotions such as discomfort and stress, can be used as an important marker to grade the ambient air quality of a region at the population level. Most studies on health impact of air pollution have focused on metropolitan districts. Considering that South Korea (hereafter Korea) is relatively small, and is significantly affected by transboundary air pollutants, research on air pollution must be conducted at a regional level, including rural areas. Furthermore, the fact that rural areas with a large proportion of elderly persons may experience relatively more damage due to air pollution, must be considered.

The Community Health Survey (CHS) is a highly representative health behavior survey that is conducted every year upon 253 administrative districts in Korea, and provides useful data for establishing community health plans. It uses a systematic survey method in addition to strict control every year, and provides highly objective data [8]. However, since domestic data are limited to certain administrative districts such as large cities, analysis of air pollution data coupled with health data at the regional level can be difficult. To overcome this limitation, data obtained by assessing air pollution level across the country including regions without measurement points are needed. This study used air pollution exposure data obtained for each administrative unit using methods identical to that of the CHS, to evaluate the effect of air pollution on subjective stress levels.

## MATERIALS AND METHODS

### Data source

In the present study, data from the CHS were used to investigate the effects of air pollution on stress levels. The CHS is an annual statistical survey (conducted since 2008), upon 253 administrative districts across the country, among all residents aged 19 years or older across all cities, counties, and districts, obtained through primary (sample location) and secondary (sample household) probability sampling. The CHS consists of both an individual and a household survey. It is used to evaluate the regional level of health based on health behavior, disease onset, medical service use, education, and economic activities, and provides highly objective community health statistics conducive to the improvement and development of community health care [9].

There are currently no CHS data regarding air pollution exposure at a city, county, or district level. To overcome this limitation, the Korean Air Quality Forecasting System (KAQFS) obtained data through a three-dimensional atmospheric chemical transport model, designed per city, county, and district in place of available atmospheric data. The chemical transport model largely consists of weather, emission, and chemical transport modeling. In terms of the weather modeling, a weather research forecasting model was used to produce three-dimensional data on wind, temperature, and humidity per unit of time, and per lattice. The resulting weather data were used in both output and chemical transport modeling. In output modeling, the Sparse Matric Operator Kernel Emissions data provided by the US Environmental Protection Agency were used to quantify the outputs per chemical species, unit of time, and space, to be used in air quality modeling after considering weather data, and the characteristics of the emission source. In chemical transport modeling, the Community Multiscale Air Quality (CMAQ) model was used. Weather factors including weather data, and the outputs quantified per chemical species, unit of time, and space by the output model were used to numerically solve the three-dimensional advection-diffusion equation, and to calculate the concentration of air pollutants per unit of time in a three-dimensional space. Setting an initial field is crucial for improving the accuracy of a numerical forecast model. Existing chemical transport models do not use domestic data, but rather use the results obtained from a model of a previous time as the initial field. Thus, data assimilation was used to make use of measurements within the radius of influence around a single lattice point, when measurements were irregularly distributed on the lattices, to adjust the model value (initial field). Data assimilation was applied to the chemical transport model to calculate the concentration of air pollutants. Results were observed at a lattice unit of 3 km× 3 km for metropolitan areas, and 9 km× 9 km for all other regions. Weighted means were calculated for each of the area of 253 healthcare administrative units representing cities, counties, and districts. We made use of the modeling data obtained for the 253 administrative districts to calculate the annual concentrations of NO_2_ and PM_10_ for each region, and matched them with the area of residence, and address of the subjects to combine the data. Modeling data of the year 2013, which are currently available, were used.

### Measurement of variables

In this study, subjective stress levels under the ‘psychological health’ domain of the CHS were used as the dependent variable. Subjects could rate their subjective stress levels as ‘very highly perceived’, ‘highly perceived’, ‘somewhat perceived’, and ‘not perceived at all’. All subjects were divided into men and women, and stratified by age into those aged < 30, 30-64, and ≥ 65 years. Total annual household income, educational level, marital status, smoking status, economic activity, average sleeping hours per day, and subjective health levels were adjusted in the model. Total annual household income, which was investigated in the household survey, was calculated as the sum of salary, real estate income, pension, and government subsidies. A natural log of this value was used as a covariate. Education levels were classified as ‘college or above’, ‘high school’, ‘no education, village school, and Chinese classics class, elementary school, and middle school’. Marital status was classified as ‘not married’, ‘living with spouse’, ‘not living with spouse’, and ‘widowed’. Smoking status was classified as ‘never smoked’, ‘used to smoke, but not smoking anymore’, ‘sometimes’, and ‘always’. Current economic activities were classified into ‘currently involved’ and ‘currently not involved’. The average sleeping hours per day was classified into ‘< 6’, ‘6-8, ‘≥ 8 hours’. The subjective level of health was classified as ‘very good’, ‘good’, ‘normal’, ‘poor’, and ‘very poor’. Subjects who either gave irrelevant responses, or refused to give responses were excluded. In total, 99,162 men, and 121,273 women, (total 220,435 subjects) were included in the analysis.

The mean annual concentrations of NO_2_, and PM_10_, mean annual temperature, and mean annual humidity were matched and combined with the subjects’ current addresses in 253 healthcare administrative districts. The subjects were divided into men and women, and NO_2_ and PM_10_ concentrations were divided into > 20 percentile. A single-pollutant model considering level of exposure as an independent variable and a multi-pollutant model adjusted for PM_10_ concentrations at different NO_2_ concentrations were analyzed. Each model was adjusted for mean annual temperature, mean annual precipitation, mean annual total household income, age, marital status, smoking status, educational level, current economic activities, subjective health status, and mean sleeping hours per day. The subjects were stratified by age into those aged < 30, 30-64, and ≥ 65 years in the analysis of the effect of exposure to air pollution on subjective stress levels.

### Statistical analysis

Subjective stress levels were rated as ‘very highly perceived’, ‘highly perceived’, ‘somewhat perceived’, and ‘almost never perceived’, and these responses were converted to scores. The odds ratios (ORs) and 95% confidence intervals (CIs) were calculated with respect to the group with the lowest level of exposure to air pollution, and to the subjects with each exposure quintile who responded, ‘almost never perceived’. A multinomial logistic regression analysis was performed since there were three dependent variables. All statistical analyses were performed using SAS version 9.4 (SAS Institute Inc., Cary, NC, USA), and R version 3.3.2 (https://cran.r-project.org/bin/windows/base/old/3.3.2/). The level of statistical significance was set at p< 0.05.

### Ethics statements

The present study was approved by the institutional review board (IRB) of Sungkyunkwan University (IRB #2018-01-011).

## RESULTS

Data from the 2013 CHS were used to investigate the effects of air pollution exposure on subjective stress levels in 99,162 men and 121,273 women. Demographic characteristics of the study participants are presented in Table 1.

**Figure 1.**
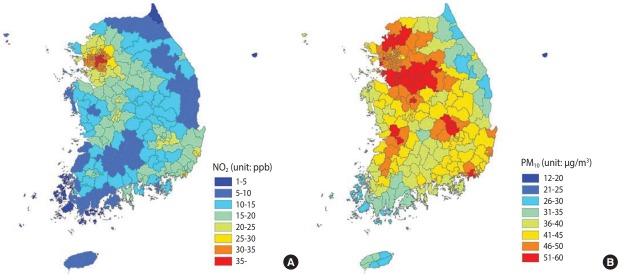
Concentration of annual average (A) nitrogen dioxide (NO2) and (B) particulate matter <10 μm in diameter (PM10) in 2013 by 253 administrative regions. Average annual concentration of the NO2 was estimated by Community Multiscale Air Quality model and synchronized by air post measurement data.

Current area of residence was classified as city, county, or district, and combined with mean annual air pollution exposure data, and divided into five percentiles. For men, the exposure intervals were < 10.46, 10.46-15.78, 15.78-19.76, 19.76-29.87, and > 29.87 parts per billion (ppb). For women, the exposure intervals were < 10.25, 10.25-15.06, 15.06-19.76, 19.76-30.08, and > 30.08 ppb. The mean annual NO_2_ and PM_10_ concentrations in 2013 were modeled at a city, county, and district level. Administrative districts corresponding to the top 10% mean annual NO_2_ concentration were mostly metropolitan areas such as Seoul Metropolitan City, Gyeonggi Province, and Incheon Metropolitan City (Figure 1).

**Figure 2.**
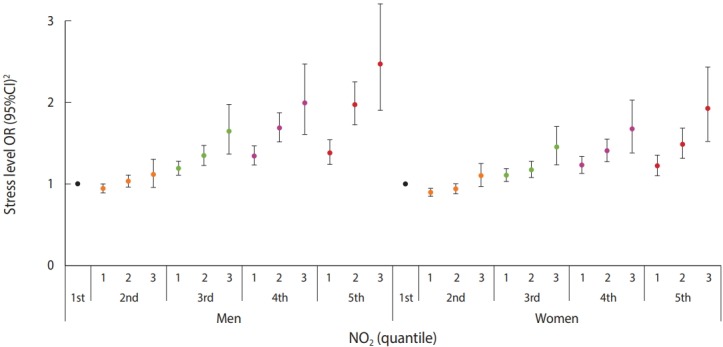
Subjective stress level in association with NO2 concentration by 20 percentiles in multi-pollutant model by gender. OR, odds ratio; CI, confidence interval; PM10, particulate matter <10 μm in diameter. 1 ORs were estimated by multinomial logistic regression adjusted for temperature, humidity, income, age, marriage, cigarette smoking, educational level, economic activity, subjective health status, sleeping hours and annual average concentration of PM10. 2 Level of subjective stress: 1, ‘I feel stress little’; 2, ‘I feel stress much’, 3: ‘I feel stress very much’ .

In the analysis of the multi-pollutant model adjusted for PM_10_ at different concentrations of NO_2_ exposure for men and women of all ages, the ORs were 1.38 (95% CI, 1.24 to 1.54) for the subjects who responded ‘almost never perceived’, 1.38 (95% CI, 1.24 to 1.54) for those who responded ‘somewhat perceived’, 1.97 (95% CI, 1.73 to 2.25) for those who responded ‘highly perceived’, and 2.47 (95% CI, 1.90 to 3.21) for those who responded ‘very highly perceived’ with respect to the OR of the subjects who responded ‘almost never perceived’ among the men subjects in the top quintile. Among the women subjects, the OR was 1.22 (95% CI, 1.10 to 1.35) for the subjects who responded, ‘somewhat perceived’, 1.49 (95% CI, 1.32 to 1.68) for those who responded, ‘highly perceived’, and 1.92 (95% CI, 1.52 to 2.43) for those who responded, ‘very highly perceived’ with respect to the OR of the women subjects who responded ‘never perceived’. Men showed higher ORs than women. As the exposure percentile increased, the OR increased (Figure 2).

The men and women subjects were divided into those aged < 30, 30-64, and ≥ 65 years to analyze the single-, and multi-pollutant models. Analysis of the single-pollutant model showed an increase in subjective stress levels as the exposure percentile increased in both men and women. In the top percentile, the OR of the men subjects who responded, ‘highly perceived’ was the highest at 2.26 (95% CI, 1.13 to 4.51), and that of the women subjects who responded the same was also the highest at 1.37 (95% CI, 0.74 to 2.53) with respect to the subjects who responded, ‘almost never perceived’. For both men and women aged 30-64 years, the OR increased as the exposure percentile increased. In the multi-pollutant model adjusted for PM_10_, the risk of subjective stress increased more significantly according to the NO_2_ concentration percentile. The OR for subjective stress was the highest among men aged 30-64 years, and this association was statistically significant (Table 2).

In the single-pollutant model in which PM_10_ concentrations were divided into quintiles with intervals of 20 percentiles, and were analyzed as an independent variable, no significant results were observed. In the subjects aged 30-64 years, the risk of subjective stress increased as the percentile increased, but this was statistically significant only for the men subjects in the top quintile (Table 3). The OR of the men subjects who responded, ‘highly perceived’ was 1.57 (95% CI: 1.33 to 1.87), and that of the women subjects who responded the same was 1.31 (95% CI: 1.11 to 1.54) with respect to the OR of the subjects who responded ‘almost never perceived’.

## DISCUSSION

Data from the 2013 CHS were used to match air pollution data with that of the subjects’ area of residence, and address across 253 healthcare administrative districts to analyze subjective stress levels. The analysis showed the OR of subjective stress increased as the air pollution level percentile increased for socioeconomically active men and women aged 30-64 years.

The CHS investigates annual history of being diagnosed of hypertension, diabetes, hyperlipidemia, stroke, myocardial infarction, arthritis, and acute and chronic diseases. Diseases have a significant impact on the subjective stress levels perceived in daily life, as well as QoL [10]. The perceived health status in the presence of activity-limiting diseases, such as arthritis, is possibly very low. In the 2013 survey, 55,632 subjects were diagnosed with hypertension, 20,922 with diabetes, and 31,675 with arthritis. Analysis of these subjects by gender, and age resulted in a small number for each stratum, which did not show statistically significant results. Therefore, the analysis was adjusted for the ratings of the subjective health status (‘very good’, ‘good’, ‘normal’, ‘poor’, and ‘very poor’) as a complex marker associated with disease onset.

Studies on exposure to air pollutants, and their effect on health from a public health perspective have focused on single air pollutants. However, since humans are exposed to various sources of air pollutants, the multi-pollutant model approach is recommended [11]. Therefore, we analyzed a single-pollutant model of NO_2_, which has direct effect on the human body, and a multi-pollutant model adjusted for PM_10_, and compared the results thereof. Men and women were divided into quintiles according to NO_2_ concentrations. The OR for subjective stress level increased as the percentile increased, and men showed higher ORs than women. Subjective stress levels are associated with the perceived QoL of an individual. A Japanese study conducted in 2005 reported a lower health-related QoL in a population group that resided near streets with large traffic volume [12]. This suggests that shortterm exposure to high levels of NO_2_ due to large traffic volume can cause acute conditions such as allergic rhinitis [13], asthma [14], worsening of respiratory symptoms [15], and can affect subjective health status such as discomfort and health-related QoL. Air pollutants inhaled by the body directly act as prooxidants or free radicals on lipids and proteins, causing oxidative stress and inducing an inflammatory response [16]. When the level of free radicals increases inside the body, atherosclerosis, heart attack, chronic inflammatory diseases, and disorders of the central nervous systems such as Parkinson’s disease, and Alzheimer’s disease can result due to this oxidative stress [17]. These conditions can lead to physical and psychological functional losses in activities of daily life.

With the analysis of men and women according to sociodemographic age, the subjects were divided into those aged < 30, 30-64, and ≥ 65 years. Men and women aged 30-64 years are the most socioeconomically active, and begin to undergo physical functional loss relative to those aged < 30 years. In all age groups, the OR of subjective stress level increased as the percentile of NO_2_ concentration increased. In the multi-pollutant model, the OR increased especially significantly in men aged 30-64 years. People in this age group are generally in the most socioeconomically active period of their lives, and are easily exposed to indoor and outdoor air pollution in daily life. Moreover, since those aged 30- 64 years responded more sensitively to NO_2_ concentration compared with other age groups due to the socioeconomic status, this group may be a more sensitive one than the other age groups. In the analysis of stress levels according to subjective health status as a complex marker of disease onset, there was a clear increase in the OR as the subjective health status worsened. Analysis of stress levels according to mean sleeping hours per day showed a clear increase in the OR as the mean sleeping hours per day decreased in men and women aged 30-64 years.

In the present study, the subjects were divided into quintiles according to NO_2_ levels, to investigate the effect of PM_10_. In the single-pollutant model, the OR of subjective stress clearly increased as the PM_10_ percentile increased in men aged 30-64 years, but the increase was statistically significant only in the 4th and 5th quintiles. Although the same association was observed among the women, it was statistically significant only in the 5th quintile (Table 3). This shows that NO_2_ has a greater impact on humans than PM_10_. Exhaust gas from vehicles on the street is one of the main causes of air pollution in Korea. Analysis of the correlation between traffic volume, and air pollutants showed a higher correlation with NO_2_ than PM_10_¸ meaning that PM_10_ concentrations do not directly reflect the influence of exhaust gas from vehicles [18]. We use various means of transportation in our daily life, and are indirectly and directly exposed to the gas exhausted by the large volume of traffic. Therefore, regulations regarding means of transportation and various sources of air pollutant are needed to reduce physical and psychological damage caused by NO_2_ exposure.

This study took advantage of the fact that the CHS provides data for each of the 253 administrative districts, and combined and analyzed the air pollutant concentration data of 253 regions. However, the CHS data, which are available to the public, do not provide the dates on which the survey was conducted, and therefore, we had to calculate mean annual air pollutant concentration to obtain values corresponding to relatively long-term exposure. Short-term exposure to air pollutants causes oxidative damage inside the body [19]. Exposure to high concentrations of air pollutants can lead to increased levels of cortisol, and norepinephrine, which are hormones released during an acute stress response [20]. Therefore, by combining the data from each day, a greater influence of short-term exposure to air pollutants, according to the date of survey, may be observed. Although the CHS data from 2013 had to be used as they were the only available data regarding air pollutant concentration, the effect of annual changes in air pollutant concentrations on health may be analyzed once data for other years are established in the future. Furthermore, the air pollution exposure data used in this study are based on outdoor air pollution. For this reason, the effect of exposure at home and the workplace, which has a significant impact, was not reflected in our results. The CHS, which is conducted by the Korea Centers for Disease Control and Prevention, commences at the end of August, and terminates at the end of October, annually. Therefore, it is conducted for 2-3 months during the year. Therefore, although mean annual NO_2_ concentration data of 253 administrative districts were combined and analyzed together, seasonal effects were not considered.

Most domestic studies on effect of air pollution on health have used atmospheric data collected by the domestic automatic air pollution measurement network. The air pollutant concentration data collected by this network lacks accuracy in regions with low densities of measurement points, and produces little data. Therefore, the KAQFS data were used to calculate the concentration of air pollutants for each administrative district in the CHS, and levels of stress due to exposure to air pollution were analyzed per city, county, and district to overcome the limitations of previous studies. However, the air pollution exposure values used in this study were not real measurements, rather, they were obtained from the CMAQ model. Although real measurements provided by the National Institute of Environmental Research in 2008 were used in the modeling process, measurements for only 226 administrative districts were used, and therefore, the model did not fully reflect the total output of air pollutants across the country.

Whereas previous domestic studies have focused on markers of disease onset and incidence associated with air pollution, this study analyzed subjective emotional states experienced in daily life prior to the onset of disease. This study combined available modeling data for a single year with the CHS data, to study the effect of exposure to air pollution on subjective stress. In using regional health markers by expanding air pollution exposure data for each administrative district, the individual and regional healthrelated QoL due to exposure to air pollution at the administrative district level, may be evaluated for the establishment of health care and welfare policies.

In this study, NO_2_ concentrations, and subjective stress levels were associated with one another in men and women aged 30-64 years. The Seoul Metropolitan City had the highest mean annual NO_2_ level of 35 ppb, whereas Jeju Island had the lowest mean annual NO_2_ level of 8 ppb according to the mean annual NO_2_ measurement data of 16 metropolises and province released by the Ministry of Environment, Korea in 2013; a significant difference in the mean annual NO_2_ concentration existed among the regions. This suggests that large differences in emotional states, which can affect disease onset, depending on the influence of socioeconomic factors at a group level, may also exist. Using the CHS data, which are obtained for each administrative district every year, evidencebased health policies may be established according to the regional levels of air pollution exposure.

## Figures and Tables

**Table 1.** Characteristics of the study subjects

Characteristics	Men (n=99,162)	Women (n=121,273)
NO_2_ (ppb)	19.0±9.4	19.0±9.4
PM_10_ (μg/m^3^)	43.2±6.4	43.2±6.4
Temperature (°C)	12.3±1.6	12.3±1.6
Humidity (%)	72.3±5.0	72.2±5.0
Income (104 KRW)	3,769.4±2,871.6	3,505.1±2,897.0
Age (yr)		
<30	11,013 (11.1)	12,880 (10.6)
30-64	63,746 (64.3)	73,690 (60.8)
≥65	24,403 (24.6)	34,703 (28.6)
Marriage		
Not married	17,848 (18.0)	14,555 (12.0)
Married	73,176 (73.8)	77,598 (64.0)
Divorced or death of a spouse	8,138 (8.2)	29,120 (24.0)
Cigarette smoking		
Never	24,224 (24.4)	114,681 (94.6)
Past	33,971 (34.3)	2,905 (2.4)
Current	40,967 (41.3)	3,687 (3.0)
Educational level		
College or above	39,157 (39.5)	34,795 (28.7)
High school	31,373 (31.6)	31,819 (26.2)
Middle school or below	28,632 (28.9)	54,659 (45.1)
Economic activity		
Yes	76,407 (77.1)	62,691 (51.7)
No	22,755 (22.9)	58,582 (48.3)
Subjective health status		
Very good	7,677 (7.7)	5,202 (4.3)
Good	36,430 (36.7)	35,855 (29.6)
Usual	38,495 (38.8)	49,787 (41.1)
Bad	12,817 (12.9)	23,298 (19.2)
Very bad	3,743 (3.9)	7,131 (5.8)
Sleeping hours (hr/d)		
<6	21,454 (21.6)	27,349 (22.6)
6–8	62,585 (63.1)	71,447 (58.9)
≥8	15,123 (15.3)	22,477 (18.5)

Values are presented as mean±standard deviation or number (%). NO_2_ , nitrogen dioxide; ppb, parts per billion; PM_10_, particulate matter <10 μm in diameter; KRW, Korean won.

**Table 2.** Subjective stress level in association with 5 quintiles for NO_2_ stratified by gender and age in single, and multi-pollutant model^1^

Age (yr)	NO_2_ (percentile)^2^	Stress level^3^	Single-pollutant		Multi-pollutant^4^	
			Men	Women	Men	Women
< 30	1st		1.00 (reference)	1.00 (reference)	1.00 (reference)	1.00 (reference)
	2nd	1	1.01 (0.82, 1.24)	0.95 (0.76, 1.19)	1.09 (0.87, 1.36)	0.99 (0.77, 1.28)
		2	1.03 (0.80, 1.34)	1.04 (0.80, 1.35)	1.10 (0.83, 1.46)	1.07 (0.80, 1.42)
		3	1.42 (0.83, 2.45)	1.39 (0.87, 2.22)	1.35 (0.75, 2.42)	1.60 (0.96, 2.68)
	3rd	1	1.11 (0.90, 1.37)	0.91 (0.72, 1.14)	1.27 (0.98, 1.64)	0.97 (0.73, 1.30)
		2	1.18 (0.92, 1.52)	0.96 (0.74, 1.24)	1.31 (0.96, 1.80)	1.00 (0.72, 1.39)
		3	1.50 (0.89, 2.53)	1.21 (0.77, 1.91)	1.37 (0.72, 2.59)	1.53 (0.86, 2.71)
	4th	1	1.21 (0.97, 1.52)	1.00 (0.78, 1.28)	1.43 (1.07, 1.91)	1.09 (0.78, 1.51)
		2	1.36 (1.04, 1.79)	1.25 (0.94, 1.65)	1.55 (1.09, 2.21)	1.31 (0.90, 1.90)
		3	2.18 (1.27, 3.73)	1.37 (0.84, 2.23)	1.96 (0.97, 3.93)	1.81 (0.95, 3.44)
	5th	1	1.12 (0.83, 1.50)	0.90 (0.65, 1.24)	1.32 (0.93, 1.88)	0.98 (0.66, 1.46)
		2	1.50 (1.05, 2.15)	1.19 (0.83, 1.71)	1.72 (1.12, 2.64)	1.26 (0.80, 1.96)
		3	2.26 (1.13, 4.51)	1.37 (0.74, 2.53)	2.02 (0.88, 4.62)	1.84 (0.86, 3.94)
30-64	1st		1.00 (reference)	1.00 (reference)	1.00 (reference)	1.00 (reference)
	2nd	1	0.95 (0.89, 1.02)	0.91 (0.85, 0.97)	1.02 (0.94, 1.11)	0.94 (0.87, 1.01)
		2	1.04 (0.96, 1.14)	0.96 (0.88, 1.04)	1.09 (0.99, 1.20)	0.98 (0.90, 1.08)
		3	1.19 (1.00, 1.41)	1.04 (0.88, 1.23)	1.26 (1.04, 1.52)	1.06 (0.87, 1.27)
	3rd	1	1.16 (1.07, 1.25)	1.08 (1.00, 1.16)	1.30 (1.17, 1.43)	1.13 (1.03, 1.25)
		2	1.36 (1.24, 1.49)	1.15 (1.05, 1.25)	1.45 (1.29, 1.64)	1.20 (1.07, 1.35)
		3	1.77 (1.49, 2.11)	1.35 (1.14, 1.60)	1.94 (1.55, 2.44)	1.37 (1.10, 1.72)
	4th	1	1.30 (1.19, 1.42)	1.18 (1.08, 1.28)	1.49 (1.32, 1.68)	1.25 (1.12, 1.40)
		2	1.75 (1.58, 1.95)	1.33 (1.20, 1.47)	1.89 (1.65, 2.18)	1.40 (1.22, 1.61)
		3	2.12 (1.75, 2.58)	1.56 (1.28, 1.89)	2.37 (1.83, 3.08)	1.59 (1.22, 2.07)
	5th	1	1.32 (1.16, 1.50)	1.18 (1.05, 1.33)	1.51 (1.30, 1.75)	1.26 (1.09, 1.45)
		2	2.01 (1.74, 2.33)	1.38 (1.21, 1.59)	2.17 (1.83, 2.58)	1.47 (1.24, 1.73)
		3	2.61 (2.00, 3.41)	1.78 (1.37, 2.31)	2.91 (2.12, 4.01)	1.82 (1.32, 2.51)
≥65	1st		1.00 (reference)	1.00 (reference)	1.00 (reference)	1.00 (reference)
	2nd	1	0.81 (0.75, 0.87)	0.79 (0.74, 0.84)	0.84 (0.77, 0.92)	0.84 (0.78, 0.91)
		2	0.98 (0.88, 1.08)	0.81 (0.75, 0.88)	1.02 (0.90, 1.15)	0.90 (0.82, 0.99)
		3	0.92 (0.71, 1.19)	1.01 (0.86, 1.19)	0.92 (0.68, 1.25)	1.10 (0.91, 1.34)
	3rd	1	0.99 (0.90, 1.09)	0.95 (0.88, 1.04)	1.04 (0.92, 1.17)	1.06 (0.95, 1.19)
		2	1.13 (0.99, 1.29)	1.00 (0.90, 1.10)	1.20 (1.02, 1.43)	1.18 (1.03, 1.35)
		3	1.17 (0.85, 1.61)	1.30 (1.06, 1.58)	1.18 (0.79, 1.77)	1.48 (1.14, 1.92)
	4th	1	1.08 (0.96, 1.22)	1.05 (0.95, 1.18)	1.15 (0.99, 1.34)	1.20 (1.05, 1.38)
		2	1.16 (0.98, 1.37)	1.12 (0.98, 1.28)	1.26 (1.02, 1.56)	1.37 (1.16, 1.62)
		3	1.12 (0.74, 1.67)	1.33 (1.02, 1.73)	1.13 (0.68, 1.88)	1.56 (1.12, 2.18)
	5th	1	1.26 (1.06, 1.50)	1.08 (0.92, 1.26)	1.34 (1.10, 1.62)	1.23 (1.03, 1.47)
		2	1.40 (1.09, 1.78)	1.33 (1.10, 1.60)	1.50 (1.15, 1.98)	1.62 (1.31, 2.01)
		3	1.54 (0.88, 2.68)	1.64 (1.14, 2.36)	1.55 (0.83, 2.91)	1.93 (1.27, 2.92)

NO_2_, nitrogen dioxide; PM_10_, particulate matter <10 μm in diameter; ppb, parts per billion.

1 Adjusted for temperature, humidity, income, age, marriage, cigarette smoking, educational level, economic activity, subjective health status, sleeping hours.

2 Concentration of NO_2_ (unit: ppb) in men: 1st (0–20th percentile): <10.46, 2nd (20–40th percentile): 10.46-15.78, 3rd (40–60th percentile): 15.78-19.76, 4th (60–80th percentile): 19.76-29.87, 5th (80–100th percentile): >29.87; Concentration of NO_2_ in women: 1st (0–20th percentile): <10.25, 2nd (20–40th percentile): 10.25-15.06, 3rd (40–60th percentile): 15.06-19.76, 4th (60–80th percentile): 19.76-30.08, 5th (80–100th percentile): >30.08.

3 Level of subjective stress: 1, ‘I feel stress little’; 2, ‘I feel stress much’; 3, ‘I feel stress very much’.

4 Adjusted for annual average concentration of PM_10_.

**Table 3.** Subjective stress level in association with five quintiles for PM_10_ stratified by gender, age in a single-pollutant model^1^

Age (yr)	PM_10_ (percentile)^2^	Stress level^3^	Men	Women
< 30	1st		1.00 (reference)	1.00 (reference)
	2nd	1	0.92 (0.76, 1.12)	0.96 (0.78, 1.19)
		2	0.83 (0.65, 1.06)	0.98 (0.77, 1.25)
		3	1.11 (0.67, 1.84)	0.95 (0.62, 1.46)
	3rd	1	0.94 (0.77, 1.15)	0.86 (0.69, 1.07)
		2	0.86 (0.67, 1.10)	0.95 (0.74, 1.22)
		3	1.40 (0.85, 2.29)	1.23 (0.81, 1.87)
	4th	1	0.85 (0.69, 1.05)	1.05 (0.83, 1.33)
		2	0.82 (0.63, 1.06)	1.08 (0.82, 1.40)
		3	1.03 (0.61, 1.73)	1.20 (0.77, 1.87)
	5th	1	0.97 (0.80, 1.19)	0.94 (0.76, 1.16)
		2	0.95 (0.75, 1.21)	1.05 (0.82, 1.34)
		3	1.42 (0.88, 2.30)	0.95 (0.63, 1.44)
30-64	1st		1.00 (reference)	1.00 (reference)
	2nd	1	1.02 (0.95, 1.09)	0.97 (0.91, 1.04)
		2	1.04 (0.95, 1.13)	1.01 (0.93, 1.09)
		3	1.14 (0.96, 1.35)	1.05 (0.89, 1.23)
	3rd	1	0.96 (0.89, 1.04)	1.00 (0.93, 1.08)
		2	1.01 (0.92, 1.10)	1.03 (0.94, 1.12)
		3	1.41 (1.19, 1.67)	1.21 (1.02, 1.43)
	4th	1	1.00 (0.92, 1.08)	0.98 (0.90, 1.06)
		2	1.07 (0.97, 1.18)	0.99 (0.90, 1.08)
		3	1.34 (1.11, 1.61)	1.03 (0.86, 1.24)
	5th	1	1.12 (1.04, 1.22)	1.05 (0.98, 1.13)
		2	1.32 (1.21, 1.45)	1.13 (1.03, 1.23)
		3	1.57 (1.33, 1.87)	1.31 (1.11, 1.54)
≥65	1st		1.00 (reference)	1.00 (reference)
	2nd	1	0.87 (0.81, 0.94)	0.87 (0.81, 0.93)
		2	0.86 (0.77, 0.96)	0.80 (0.73, 0.87)
		3	0.78 (0.59, 1.02)	0.83 (0.70, 0.99)
	3rd	1	0.87 (0.79, 0.96)	0.82 (0.75, 0.89)
		2	0.89 (0.78, 1.02)	0.84 (0.76, 0.93)
		3	0.98 (0.73, 1.33)	1.20 (1.00, 1.44)
	4th	1	0.81 (0.73, 0.90)	0.77 (0.70, 0.85)
		2	0.96 (0.82, 1.12)	0.75 (0.67, 0.85)
		3	0.64 (0.43, 0.94)	0.90 (0.71, 1.14)
	5th	1	0.98 (0.89, 1.08)	0.99 (0.91, 1.09)
		2	1.00 (0.87, 1.15)	0.94 (0.84, 1.05)
		3	1.05 (0.76, 1.45)	0.99 (0.80, 1.24)

Values are presented as odds ratio (95% confidence interval). PM_10_, particulate matter <10 μm in diameter.

1 Adjusted for temperature, humidity, income, age, marriage, cigarette smoking, educational level, economic activity, subjective health status, sleeping hours.

2 Concentration of PM_10_ (unit: mg/m^3^) in men: 1st (0-20th percentile): <38.36, 2nd (20-40th percentile): 38.36-42.47, 3rd (40-60th percentile): 42.47-45.59, 4th (60-80th percentile): 45.59-48.45, 5th (80-100th percentile): >48.45; Concentration of PM_10_ in women: 1st (0-20th percentile): <38.36, 2nd (20-40th percentile): 38.36-42.44, 3rd (40-60th percentile): 42.44-45.47, 4th (60-80th percentile): 45.47-48.45, 5th (80-100th percentile): >48.45.

3 Level of subjective stress: 1, ‘I feel stress little’; 2, ‘I feel stress much’; 3, ‘I feel stress very much’.

## SUPPLEMENTAL MATERIALS

Supplementary Material: Korean version is available at http://www.e-epih.org/.
