# Supplementary material for: Ambient air quality and subjective stress level using Community Health Survey data in Korea
Source: Epidemiol Health. 2018 Jun 28;40:e2018028. doi: 10.4178/epih.e2018028 (PMC6178364; doi:10.4178/epih.e2018028)
Supplement: Supplementary file 1 [file epih-40-e2018028-supplementary.pdf]

Volume: 40, Article ID: e2018028

https://doi.org/10.4178/epih.e2018028

# Ambient air quality and subjective stress level using Community Health Survey data in Korea

Myung-Jae Hwang[1], Hae-Kwan Cheong[1], Jong-Hun Kim[1], Youn-Seo Koo[2], Hui-Young Yun[2]

[1]Department of Social and Preventive Medicine, Sungkyunkwan University School of Medicine, 2066 Seobu-ro Jangan-gu, Suwon, Gyeonggi-do 16419, Republic of Korea

[2]Department of Environmental and Energy Engineering, Anyang University, Republic of Korea

**Address for correspondence:**

Myung-Jae Hwang

Department of Social and Preventive Medicine

Sungkyunkwan University School of Medicine

2066 Seobu-ro, Jangan-gu, Suwon, Gyeonggi-do 16419, Republic of Korea

Tel: +82 31 299 6295

Fax: +82 31 299 6299

Email: mj6663@naver.com

Volume: 40, Article ID: e2018028

https://doi.org/10.4178/epih.e2018028

**Abstract**

**Objectives**

대기오염은 노출된 인구집단에 다양한 질병을 발생시키며 이환으로 진행되기 전에도 신체적 및 정신적 기능 감소를 야기할 수 있다. 본 연구에서는 대기오염으로 인해 일상 생활에서 정신적으로 느끼는 주관적 스트레스의 연관성을 분석하고자 한다.

**Methods**

2013년 지역사회건강조사 대상자 남성 99,162명, 여성121,273명을 대상으로 현재 거주지에 따라 253개 보건행정지역단위로 Korean Air Quality Forecasting System (KAQFS) 대기물질농도 모델링자료를 결합하여 주관적 스트레스 정도에 따라 5분위로 범주화하여 다항로지스틱회귀을 하였다. 노출수준을 연평균 이산화질소(nitrogen dioxide, $NO_2$)농도에 따라 5분위수로 나누어, $NO_2$ 농도만을 이용한 단일오염물질모형과 미세먼지(particulate matter, $PM_{10}$)농도를 보정한 복합오염물질모형으로 나누어 분석하였다.

**Results**

대상자를 남성과 여성으로 나누어 분석한 결과, 복합오염물질모형에서 30세 이상 65세 미만의 남성과 여성에서 가장 높은 $NO_2$ 농도 분위에서 스트레스 수준의 오즈비가 가장 높았다(남성: 2.91 (95%CI: 2.12-4.01), 여성: 1.82 (95% CI: 1.32-2.51)). $NO_2$ 농도 분위가 높아질수록 오즈비가 증가하였고, 모든 계층에서 남성이 여성보다 오즈비가 더 컸다.

**Conclusions**

본 연구에서는 연평균 $NO_2$ 농도와 주관적 스트레스 정도가 관련이 있는 것으로 나타났다. 특히, 사회 경제적 활동이 활발한 30 세 이상 65 세 미만의 남성과 여성에서 관련성이 뚜렷하게 나타났다.

**Key words:** air pollution, ambient air quality, stress, quality of life, Community Health Survey

Volume: 40, Article ID: e2018028

https://doi.org/10.4178/epih.e2018028

**Introduction**

세계적으로 급속한 산업화와 도시화로 인한 대기오염이 심해지고 있으며 이로 인한 건강피해도 증가하고 있다. 건강영향을 초래하는 대기오염의 주원인은 에너지산업, 자동차 등으로 대표되는 화석연료의 연소에서 기인한 것으로 대표적인 대기오염물질로 미세먼지(particulate matter, PM), 이산화질소(nitrogen dioxide, $NO_2$), 일산화탄소(carbon monoxide, CO), 오존(ozone, $O_3$) 등이 있다. 인체의 호흡기를 통해 흡입된 대기오염 물질은 폐 세포의 산화손상을 일으켜 폐질환, 기관지염 등을 유발하고 장기노출로 인한 지속적인 손상은 폐암을 유발한다. 흡입된 입자는 혈류를 타고 전신 염증반응을 일으켜 혈관과 혈액응고 작용에도 영향을 주게 되어 자율신경계에 급성 영향을 미칠 수 있다 [1]. 대기오염물질은 당뇨와 고혈압과 같은 대사질환이나 심혈관질환을 악화시킬 뿐 아니라 최근에는 우울증, 자폐 스펙트럼 장애, 알츠하이머병 및 파킨슨병과 같은 신경퇴행성질환에도 영향을 미친다고 보고되고 있다[2,3,4,5]. 2012년, 세계보건기구(World Health Organization, WHO)는 세계적으로 대기오염을 포함한 공기오염으로 인한 사망자 수가 연간 약 7백 만 명에 이를 것으로 추산하였는데, 이는 전체 사망 인구의 약 12.5%에 해당된다[6]. 대기오염은 공중보건의 주요 위험요인 중 하나로 부각되고 있으며 개발도상국뿐만 아니라 선진국에서도 건강피해로 인한 지속적인 보건학적 문제를 야기하며 관심이 커지고 있다. 국내외에서 대기오염 노출로 인한 사망 및 질병 발생에 대한 다양한 연구가 진행되고 있다.

그러나 대부분의 연구들은 대기오염 노출로 인한 사망, 질환 발생 및 이환과 같은 지표에 집중되어 있다. 인체는 대기오염에 노출되면 건강관련 질환이 발생하거나 및 이환 수준으로 진행되기 전에도 신체적 및 정신적 기능 감소가 야기될 수 있다. 이러한 영향은 일상생활에서 느끼는 감정상태에 반영될 수 있고, 불쾌감, 스트레스, 성가심, 분노, 나아가 건강관련 삶의 질 수준까지도 영향을 미칠 수 있다. 2000년, SAPALDIA (The Swiss Study on Air Pollution and Lung Diseases in Adults)팀에서 9,651명을 대상으로 $PM_{10}$과 $NO_2$농도에 따른 'annoyance (성가심)'를 점수화 하여 연관성을 분석한 결과, 노출 농도가 높아질수록 점수가 높아지는 강한 상관관계를 보였다($r>0.85$) [7]. 이처럼 대기오염 노출은 개인의 주관적 감정상태에 영향을 미칠 수 있다. 역학적으로 대기오염 노출에 따라 불쾌감, 스트레스 등의 주관적 및 객관적인 감정척도 간의 연관성은 집단의 수준에서 지역 내 대기질 등급을 매기는 중요한 지표로 이용될 수 있다. 기존 대기오염 건강영향 연구들은 대부분 측정점을 기준으로 하여 대도시지역에 국한된 연구를 수행하였다. 상대적으로 면적이 좁고 월경성 오염물질의 영향이 높은 국내 대기오염의 특성을 감안할 때,

Volume: 40, Article ID: e2018028

https://doi.org/10.4178/epih.e2018028

대기오염 연구에 있어서 농촌지역을 포함한 전국적인 차원의 연구가 필요하다. 특히 노령인구의 분율이 높은 농촌지역은 대기오염의 피해가 상대적으로 클 수 있는 점을 감안하여야 한다.

지역사회건강조사는 253개 보건행정단위를 기준으로 매년 시행되는 대표성이 뛰어난 건강행태조사로써 지역보건의료계획을 수립하는 데 유용한 자료로 활용되고 있다. 매년 높은 수준의 조사 질 관리와 체계화된 조사 방법으로 시행되고 있고, 객관성 높은 자료로 이용된다[8]. 그러나 국내 기존 측정자료는 행정지역을 기준으로 볼 때 대도시 등 일부지역에 국한되어 있으므로 전국적인 수준에서 대기오염 자료와 건강자료를 연계하여 분석하는데 어려움이 있었다. 이를 극복하기 위해 측정점이 없는 지역을 포함한 전국의 대기오염도를 동일한 방법으로 평가한 자료가 필요하다. 본 연구에서는 전국을 대상으로 동일한 지역별로 산출한 대기오염 노출 자료를 이용하여 연평균 대기오염 농도에 따른 주관적 스트레스 수준의 영향을 평가하고자 하였다.

Volume: 40, Article ID: e2018028

https://doi.org/10.4178/epih.e2018028

## Materials and Methods

### Data source

본 연구에서는 대기오염 노출로 인한 스트레스의 영향을 분석하기 위해 매년 전국에서 253 개 보건행정단위를 기준으로 시행되는 지역사회건강조사 통계자료를 이용하였다. 지역사회건강조사는 2008 년 이후 1 차(표본지점), 2 차(표본가구)를 통해 확률표본 추출된 만 19 세 이상의 모든 주민을 대상으로 전국 시・군・구에서 시행되는 통계조사이다. 지역사회건강조사는 크게 개인 설문조사와 가구 설문조사로 구성되어 있고, 건강행태, 이환, 의료이용, 교육 및 경제활동 등 지역간 건강수준을 평가하고 객관성 높은 지역보건통계를 산출하여 지역보건의료의 개선과 발전을 위해 좋은 자료로 사용되고 있다[9].

현재 국내에는 지역사회건강조사의 보건소 관할 시・군・구 단위의 대기오염 노출 데이터가 없다. 이러한 한계점은 보완하기 위해 본 연구의 대기오염 노출 데이터로는 국내의 기존 대기환경자료 대신 시・군・구 단위로 산출된 3 차원 대기화학수송모델을 이용하여 산정된 Korean Air Quality Forecasting System (KAQFS) 자료를 이용하였다. 화학수송모델은 크게 기상모델링, 배출량모델링, 화학수송모델링으로 구성되어 있다. 기상모델링은 WRF (Weather Research Forecasting) 모델을 이용하여 시간별, 격자별로 바람장, 온・습도장을 비롯한 3 차원자료를 생성하였고, 생성된 기상자료를 배출량모델링과 화학수송모델링의 입력자료로 이용하였다. 배출량 모델링은 미국 환경보호청(US EPA)에서 제공하는 SMOKE (Sparse Matrix Operator Kernel Emissions)를 이용하여 기상자료와 각 배출원의 특성을 고려하여 대기질 모델링에 적용할 수 있는 화학종별, 시간별, 공간별 배출량을 생성하였다. 화학수송모델링에는 CMAQ (Community Multiscale Air Quality)모델을 이용하였다. 기상자료의 기상인자와 배출량 모델에서 산정된 화학종별, 시간별, 공간별 배출량을 활용하여 3 차원 이류확산방정식을 수치적으로 풀어 3 차원 공간상에서 매 시간별 오염물질농도를 계산하였다. 수치예보 모델에서 정확도를 개선하기 위해서는 초기장을 설정하는 것이 매우 중요한데, 기존 화학수송모델은 국내 측정자료를 활용하지 않고 이전 시간의 모델 산출 결과를 그대로 초기장으로 사용하고 있다. 따라서, 자료동화방법을 적용하여 모델링 동격자상에 불규칙적으로 분포한 관측치들이 존재하는 경우에 하나의 모델 값 격자점을 둘러싼 영향 반경 내 존재하는 측정치를 이용하여 모델 값(초기장)을 보정하였다. 최종적으로 화학수송모델에 자료동화방법을 적용하여 대기오염 물질농도를 계산하였다. 산출된 결과는 수도권의 경우 3 km X 3 km, 그외 지역은 9 km X 9 km 격자 단위로 생성하였다. 이렇게 생성된

Volume: 40, Article ID: e2018028

https://doi.org/10.4178/epih.e2018028

결과는 253 개 시군구의 경계영역에 따라 각 행정구역별 가중평균치를 산출하여 적용하였다. 우리는 253 개 보건행정단위로 산출된 모델링자료를 이용하여 각 지역의 연평균 $NO_2$ 와 $PM_{10}$ 농도를 산정하여 대상자의 거주지 주소와 매칭하여 데이터를 결합하였다. 현재 가용한 2013 년 모델링자료를 이용하였다.

Measurement of variables

본 연구에서는 종속 변수로 ‘정신건강’ 영역에 해당하는 주관적 스트레스 수준 문항을 이용하였다. 주관적 스트레스 수준은 ‘대단히 많이 느낀다’, ‘많이 느끼는 편이다’, ‘조금 느끼는 편이다’, ‘거의 느끼지 않는다’로 4 단계 문항으로 되어 있다. 전체 대상자를 남성, 여성으로 나누어 30 세 미만, 30 세 이상 65 세 미만, 65 세 이상으로 층화 하였고, 연간 가구 총소득, 최종학력, 혼인상태, 흡연상태, 경제활동 유무, 하루 평균 수면시간, 주관적 건강수준을 보정하였다. 가구 조사 문항 중 연간 가구 총소득은 임금, 부동산소득, 연금이자, 정부 보조금 등 모든 수입을 합쳐 최근 1 년 동안 가구의 총 소득(단위: 만원)을 산출한 값으로 자연로그를 취하여 보정변수로 하였다. 최종학력은 ‘대학교 이상’, ‘고등학교 졸업’, ‘무학, 서당 및 한학, 초등학교, 중학교 졸업’ 로 분류하였다. 혼인상태는 ‘미혼’, ‘배우자가 있으며 함께 살고 있음’, ‘배우자가 있으나 함께 살고 있지 않음, 배우자 사망으로 인한 배우자가 없음, 이혼으로 배우자가 없음’ 로 분류하였다. 흡연상태는 ‘피운 적 없음’, ‘과거에는 피웠으나 현재 피우지 않음’, ‘가끔 피움, 매일 피움’ 로 분류하였다. 현재 경제활동 유무는 ‘예’, ‘아니오’ 로 분류하였고, 하루 평균 수면시간은 ‘6 시간 미만’, ‘6 시간 이상 8 시간 미만’, ‘8 시간 이상’ 로 분류하였다. 주관적 건강수준은 응답보기에 따라 ‘매우 좋음’, ‘좋음’, ‘보통’, ‘나쁨’, ‘매우 나쁨’ 로 분류하였다. 각 변수에 따라 비해당, 응답거부에 응답한 대상자를 제외한 결과, 남성 99,162 명, 여성 121,273 명으로 총 220,435 명을 대상으로 분석하였다.

우리는 조사대상자의 현재 거주지 주소의 253 개 보건행정 단위 변수를 이용하여 연평균 $NO_2$, $PM_{10}$, 연평균기온, 연평균습도를 매칭하여 결합하였다. 전체 대상자를 남성, 여성으로 분류하여 각 그룹에서 $NO_2$ 농도와 $PM_{10}$ 농도를 20 percentile 에 따라 5 분위로 나누었다. 남성과 여성 전체 대상자의 각 그룹에서 $NO_2$ 과 $PM_{10}$ 농도를 5 분위로 나누어 노출수준을 독립변수로 고려한 single-pollutant model 과 독립변수로 $NO_2$ 노출에 따른 $PM_{10}$ 농도를 보정한 multi-pollutant model 로 나누어 분석하였다. 각 model 에서 연평균 기온, 연평균 강수량, 연간 가구 총소득, 연령, 혼인상태, 흡연상태, 최종학력, 현재 경제활동 여부, 주관적 건강수준, 하루 평균 수면시간을 보정하여 분석을 시행하였다. 남, 여

Volume: 40, Article ID: e2018028

https://doi.org/10.4178/epih.e2018028

그룹에서 인구사회학적 연령에 따라 30 세 미만, 30 세 이상 65 세 미만, 65 세 이상으로 분류하여 대기오염 노출로 인한 주관적 스트레스 수준을 분석하였다.

Statistical analysis

주관적 스트레스 상태는 정도에 따라 '대단히 많이 느낀다', '많이 느끼는 편이다', '조금 느끼는 편이다', '거의 느끼지 않는다' 4 가지 단계로 분류되어 있고, 이를 범주화하였다. 대기오염 노출 농도가 가장 낮은 그룹을 기준으로 각 노출 분위에서 '거의 느끼지 않는다'에 응답한 대상자를 기준으로 오즈비(odds ratio, OR)를 산출하였다. 종속변수가 3 개 이상인 경우이므로 다항로지스틱회귀분석(multinomial logistic regression)을 시행하였다. 본 연구에서 통계분석은 SAS 9.4 와 R version 3.3.2 를 이용하였고, 95% 신뢰구간을 제시하였다. 유의수준은 0.05 로 설정하여 통계검정하였다.

IRB

본 연구는 성균관대학교 연구윤리위원회의 (IRB #2018-01-011) 심사 승인을 받았다.

## Results

2013 년 지역사회 건강조사를 이용하여 대기오염 노출로 인한 주관적 스트레스 정도를 보기 위해 남성 99,162 명, 여성 121,273 명을 대상으로 분석하였다. 연구 대상자들의 인구특성에 대한 결과를 제시하였다 (Table 1).

**Table 1. Characteristics of the study subjects (continued)**

| **Variables** | **Sex** | **Male** | | **Female** | |
|---|---|---|---|---|---|
| | | **Mean** | **SD**[†] | **Mean** | **SD**[†] |
| **$NO_2$ (ppb)** | | 19.0 | 9.4 | 19.0 | 9.4 |
| **$PM_{10}$ (㎍/㎥)** | | 43.2 | 6.4 | 43.2 | 6.4 |
| **Temperature (℃)** | | 12.3 | 1.6 | 12.3 | 1.6 |
| **Humidity (%)** | | 72.3 | 5.0 | 72.2 | 5.0 |
| **Income (10,000 KRW)** | | 3769.4 | 2871.6 | 3505.1 | 2897.0 |
| | | **N** | **%** | **N** | **%** |
| **Total** | | 99,162 | 100.0 | 121,273 | 100.0 |
| **Age (years)** | **< 30** | 11,013 | 11.1 | 12,880 | 10.6 |
| | **30–64** | 63,746 | 64.3 | 73,690 | 60.8 |
| | **≥65** | 24,403 | 24.6 | 34,703 | 28.6 |
| **Marriage** | **Not married** | 17,848 | 18.0 | 14,555 | 12.0 |

Volume: 40, Article ID: e2018028

https://doi.org/10.4178/epih.e2018028

| | | | | | |
|---|---|---|---|---|---|
| | **Married** | 73,176 | 73.8 | 77,598 | 64.0 |
| | **Divorced or death of a spouse** | 8,138 | 8.2 | 29,120 | 24.0 |
| **Cigarette smoking** | **Never** | 24,224 | 24.4 | 114,681 | 94.6 |
| | **Past** | 33,971 | 34.3 | 2,905 | 2.4 |
| | **Current** | 40,967 | 41.3 | 3,687 | 3.0 |
| **Educational level** | **College or above** | 39,157 | 39.5 | 34,795 | 28.7 |
| | **High school** | 31,373 | 31.6 | 31,819 | 26.2 |
| | **Middle school or below** | 28,632 | 28.9 | 54,659 | 45.1 |
| **Economic activity** | **Yes** | 76,407 | 77.1 | 62,691 | 51.7 |
| | **No** | 22,755 | 22.9 | 58,582 | 48.3 |
| **Subjective health status** | **Very good** | 7,677 | 7.7 | 5,202 | 4.3 |
| | **Good** | 36,430 | 36.7 | 35,855 | 29.6 |
| | **Usual** | 38,495 | 38.8 | 49,787 | 41.1 |
| | **Bad** | 12,817 | 12.9 | 23,298 | 19.2 |
| | **Very bad** | 3,743 | 3.9 | 7,131 | 5.8 |
| **Sleeping hours (h/day)** | **<6 h** | 21,454 | 21.6 | 27,349 | 22.6 |
| | **6–8 h** | 62,585 | 63.1 | 71,447 | 58.9 |
| | **≥8 h** | 15,123 | 15.3 | 22,477 | 18.5 |

KRW, Korean Won; ppb, parts per billion; $PM_{10}$, particulate matter <10 μm in diameter; SD, standard deviation

†: Standard deviation

현재 거주지를 시・군・구로 분류하여 연평균 대기오염 노출 데이터를 결합하여 각각의 그룹에서 5 분위로 나눈 결과, 남성에서 10.46 ppb 미만, 10.46-15.78 ppb, 15.78-19.76 ppb, 19.76-29.87 ppb, 29.87 ppb 이상으로 범주화 하였고, 여성에서 10.25ppb 미만, 10.25-15.06 ppb, 15.06-19.76 ppb, 19.76-30.08 ppb, 30.08ppb 이상으로 범주화 하였다. 2013 년 연평균 $NO_2$ 와 $PM_{10}$ 의 농도를 시・군・구 단위로 모델링 한 결과, 연평균 $NO_2$ 농도가 높은 상위 10%에 해당하는 보건행정 지역은 대부분 서울특별시, 경기도, 인천광역시에 해당하는 수도권 지역이었다(Figure 1).

Volume: 40, Article ID: e2018028

https://doi.org/10.4178/epih.e2018028

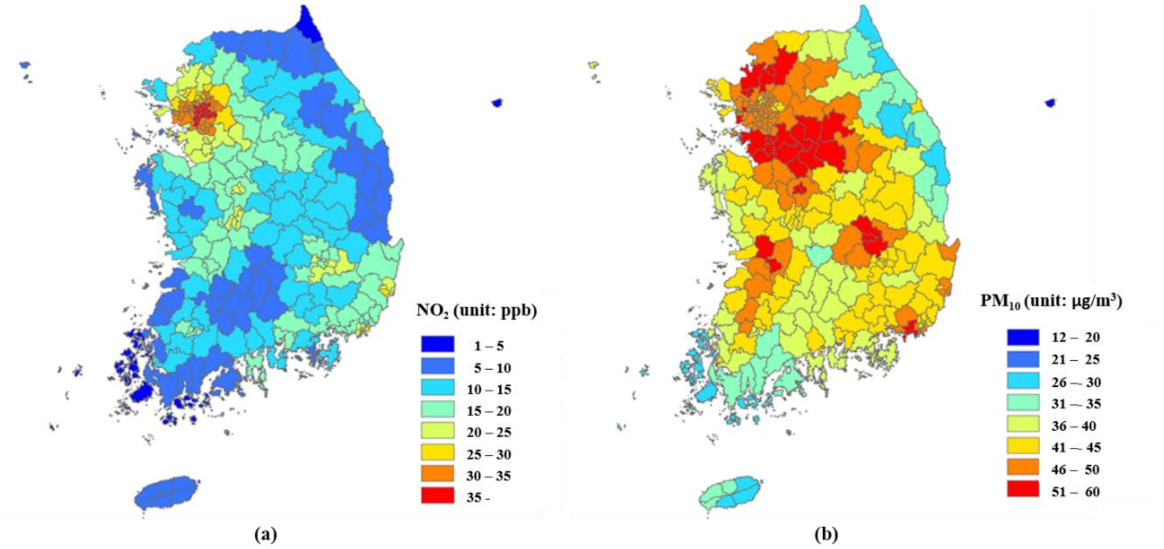


**Figure 1. Average annual concentration of (a) $NO_2$ (in ppb), and (b) $PM_{10}$ (in μg/m$^3$) in 2013 in the 253 administrative regions. Average annual concentration of $NO_2$ was estimated by the CMAQ model and synchronized by air post measurement.**

CMAQ, Community Multiscale Air Quality; $NO_2$, Nitrogen Dioxide; $PM_{10}$, particulate matter <10 μm in diameter

남성과 여성에서 전체 연령을 대상으로 $NO_2$ 노출 분위에 따라 $PM_{10}$ 을 보정한 multi-pollutant model 분석 결과, 가장 높은 5 분위에서 남성의 OR 은 ‘거의 느끼지 않는다’를 기준으로 ‘조금 느끼는 편이다’에 응답한 대상자의 OR 은 1.38 (95% CI: 1.24-1.54), ‘많이 느끼는 편이다’는 1.97 (95% CI: 1.73-2.25), ‘대단히 많이 느낀다’는 2.47 (95% CI: 1.90-3.21)이었다. 여성에서 ‘거의 느끼지 않는다’를 기준으로 ‘조금 느끼는 편이다’에 응답한 대상자의 OR 은 1.22 (95% CI: 1.10-1.35), ‘많이 느끼는 편이다’는 1.49 (95% CI: 1.32-1.68), ‘대단히 많이 느낀다’는 1.92 (95% CI: 1.52-2.43)로 남성에서 OR 이 더 높았고, 노출 분위가 증가할수록 OR 이 증가하였다(Figure 2).

Volume: 40, Article ID: e2018028

https://doi.org/10.4178/epih.e2018028

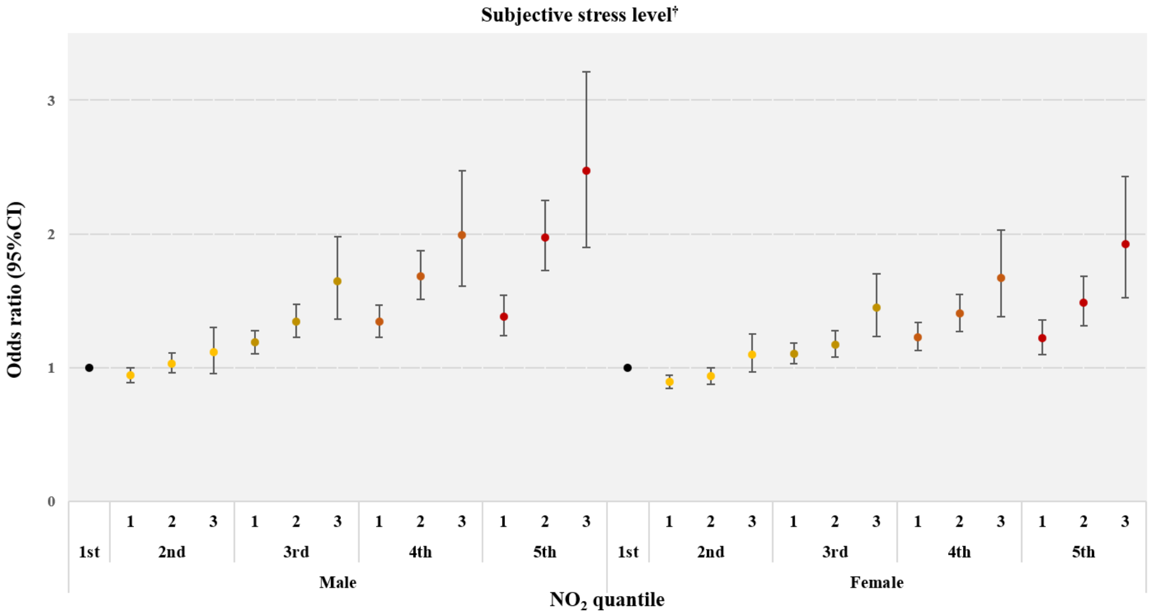


**Figure 2. Odds ratios (95% confidence interval) of subjective stress level in association with $NO_2$ concentration five quintiles in a multi-pollutant model, by sex. The ORs were estimated by multinomial logistic regression, adjusted for temperature, humidity, income, age, marriage, cigarette smoking, educational level, economic activity, subjective health status, sleeping hours, and average annual concentration of $PM_{10}$.**

†: Level of subjective stress. 1: 'I feel stress little', 2: 'I feel stress much', 3: 'I feel stress very much'

CI, confidence interval; $NO_2$, nitrogen dioxide; $PM_{10}$, particulate matter <10 μm in diameter

남성과 여성 대상자를 30 세 미만, 30 세 이상 65 세 미만, 65 세 이상으로 나누어 single-pollutant model, multi-pollutant model 로 분석하였다. Single-pollutant model 분석 결과, 30 세 미만에서 남성과 여성 모두 농도 분위가 증가할수록 스트레스 수준의 위험이 증가하였고, 가장 높은 5 분위에서 '거의 느끼지 않는다'를 기준으로 '많이 느끼는 편이다'에 응답한 남성의 OR 은 2.26 (95% CI: 1.13-4.51), 여성의 OR 은 1.37 (95% CI: 0.74-2.53)로 가장 높았다. 30 세 이상 65 세 미만의 남성과 여성 모두 농도 분위 수가 증가할수록 OR 이 증가하였다. $PM_{10}$ 을 보정한 multi-pollutant model 에서 $NO_2$ 농도 분위에 따른 스트레스 수준의 위험이 더 컸고, 특히 30 세 이상 65 세 미만의 남성에서 OR 이 가장 높았으며 통계적으로 유의한 결과를 보였다(Table 2).

Volume: 40, Article ID: e2018028

https://doi.org/10.4178/epih.e2018028

**Table 2. Odds ratios (95% confidence interval) of subjective stress level in association with 5 quintiles for $NO_2$ stratified by sex and age in single-pollutant, and multi-pollutant model (continued)**

| Age group | $NO_2$ percentile[‡‡] | level of stress[#] | Single-pollutant model | | | | Multi-pollutant model | | | |
|---|---|---|---|---|---|---|---|---|---|---|
| | | | Male | | Female | | Male | | Female | |
| | | | OR[†] | 95% CI[††] | OR[†] | 95% CI[††] | OR[†] | 95% CI[††] | OR[†] | 95% CI[††] |
| < 30 | 1st | | ref | | | | ref | | | |
| | 2nd | 1 | 1.01 | (0.82–1.24) | 0.95 | (0.76–1.19) | 1.09 | (0.87–1.36) | 0.99 | (0.77–1.28) |
| | | 2 | 1.03 | (0.80–1.34) | 1.04 | (0.80–1.35) | 1.10 | (0.83–1.46) | 1.07 | (0.80–1.42) |
| | | 3 | 1.42 | (0.83–2.45) | 1.39 | (0.87–2.22) | 1.35 | (0.75–2.42) | 1.60 | (0.96–2.68) |
| | 3rd | 1 | 1.11 | (0.90–1.37) | 0.91 | (0.72–1.14) | 1.27 | (0.98–1.64) | 0.97 | (0.73–1.30) |
| | | 2 | 1.18 | (0.92–1.52) | 0.96 | (0.74–1.24) | 1.31 | (0.96–1.80) | 1.00 | (0.72–1.39) |
| | | 3 | 1.50 | (0.89–2.53) | 1.21 | (0.77–1.91) | 1.37 | (0.72–2.59) | 1.53 | (0.86–2.71) |
| | 4th | 1 | 1.21 | (0.97–1.52) | 1.00 | (0.78–1.28) | 1.43 | (1.07–1.91) | 1.09 | (0.78–1.51) |
| | | 2 | 1.36 | (1.04–1.79) | 1.25 | (0.94–1.65) | 1.55 | (1.09–2.21) | 1.31 | (0.90–1.90) |
| | | 3 | 2.18 | (1.27–3.73) | 1.37 | (0.84–2.23) | 1.96 | (0.97–3.93) | 1.81 | (0.95–3.44) |
| | 5th | 1 | 1.12 | (0.83–1.50) | 0.90 | (0.65–1.24) | 1.32 | (0.93–1.88) | 0.98 | (0.66–1.46) |
| | | 2 | 1.50 | (1.05–2.15) | 1.19 | (0.83–1.71) | 1.72 | (1.12–2.64) | 1.26 | (0.80–1.96) |
| | | 3 | 2.26 | (1.13–4.51) | 1.37 | (0.74–2.53) | 2.02 | (0.88–4.62) | 1.84 | (0.86–3.94) |
| 30-64 | 1st | | ref | | | | ref | | | |
| | 2nd | 1 | 0.95 | (0.89–1.02) | 0.91 | (0.85–0.97) | 1.02 | (0.94–1.11) | 0.94 | (0.87–1.01) |
| | | 2 | 1.04 | (0.96–1.14) | 0.96 | (0.88–1.04) | 1.09 | (0.99–1.20) | 0.98 | (0.90–1.08) |
| | | 3 | 1.19 | (1.00–1.41) | 1.04 | (0.88–1.23) | 1.26 | (1.04–1.52) | 1.06 | (0.87–1.27) |
| | 3rd | 1 | 1.16 | (1.07–1.25) | 1.08 | (1.00–1.16) | 1.30 | (1.17–1.43) | 1.13 | (1.03–1.25) |
| | | 2 | 1.36 | (1.24–1.49) | 1.15 | (1.05–1.25) | 1.45 | (1.29–1.64) | 1.20 | (1.07–1.35) |

Volume: 40, Article ID: e2018028

https://doi.org/10.4178/epih.e2018028

| | | | | | | | | | | |
|---|---|---|---|---|---|---|---|---|---|---|
| | | **3** | 1.77 | (1.49–2.11) | 1.35 | (1.14–1.60) | 1.94 | (1.55–2.44) | 1.37 | (1.10–1.72) |
| | | **1** | 1.30 | (1.19–1.42) | 1.18 | (1.08–1.28) | 1.49 | (1.32–1.68) | 1.25 | (1.12–1.40) |
| | **4th** | **2** | 1.75 | (1.58–1.95) | 1.33 | (1.20–1.47) | 1.89 | (1.65–2.18) | 1.40 | (1.22–1.61) |
| | | **3** | 2.12 | (1.75–2.58) | 1.56 | (1.28–1.89) | 2.37 | (1.83–3.08) | 1.59 | (1.22–2.07) |
| | | **1** | 1.32 | (1.16–1.50) | 1.18 | (1.05–1.33) | 1.51 | (1.30–1.75) | 1.26 | (1.09–1.45) |
| | **5th** | **2** | 2.01 | (1.74–2.33) | 1.38 | (1.21–1.59) | 2.17 | (1.83–2.58) | 1.47 | (1.24–1.73) |
| | | **3** | 2.61 | (2.00–3.41) | 1.78 | (1.37–2.31) | 2.91 | (2.12–4.01) | 1.82 | (1.32–2.51) |
| **≥65** | **1st** | | | ref | | | | ref | | |
| | | **1** | 0.81 | (0.75–0.87) | 0.79 | (0.74–0.84) | 0.84 | (0.77–0.92) | 0.84 | (0.78–0.91) |
| | **2nd** | **2** | 0.98 | (0.88–1.08) | 0.81 | (0.75–0.88) | 1.02 | (0.90–1.15) | 0.90 | (0.82–0.99) |
| | | **3** | 0.92 | (0.71–1.19) | 1.01 | (0.86–1.19) | 0.92 | (0.68–1.25) | 1.10 | (0.91–1.34) |
| | | **1** | 0.99 | (0.90–1.09) | 0.95 | (0.88–1.04) | 1.04 | (0.92–1.17) | 1.06 | (0.95–1.19) |
| | **3rd** | **2** | 1.13 | (0.99–1.29) | 1.00 | (0.90–1.10) | 1.20 | (1.02–1.43) | 1.18 | (1.03–1.35) |
| | | **3** | 1.17 | (0.85–1.61) | 1.30 | (1.06–1.58) | 1.18 | (0.79–1.77) | 1.48 | (1.14–1.92) |
| | | **1** | 1.08 | (0.96–1.22) | 1.05 | (0.95–1.18) | 1.15 | (0.99–1.34) | 1.20 | (1.05–1.38) |
| | **4th** | **2** | 1.16 | (0.98–1.37) | 1.12 | (0.98–1.28) | 1.26 | (1.02–1.56) | 1.37 | (1.16–1.62) |
| | | **3** | 1.12 | (0.74–1.67) | 1.33 | (1.02–1.73) | 1.13 | (0.68–1.88) | 1.56 | (1.12–2.18) |
| | | **1** | 1.26 | (1.06–1.50) | 1.08 | (0.92–1.26) | 1.34 | (1.10–1.62) | 1.23 | (1.03–1.47) |
| | **5th** | **2** | 1.40 | (1.09–1.78) | 1.33 | (1.10–1.60) | 1.50 | (1.15–1.98) | 1.62 | (1.31–2.01) |
| | | **3** | 1.54 | (0.88–2.68) | 1.64 | (1.14–2.36) | 1.55 | (0.83–2.91) | 1.93 | (1.27–2.92) |

CI, confidence interval; $NO_2$, nitrogen dioxide; OR, odds ratio; $PM_{10}$, particulate matter <10 μm in size; ppb, parts per billion

†: Odds ratio (adjusted for temperature, humidity, income, age, marriage, cigarette smoking, educational level, economic activity, subjective health status, sleeping hours)

††: 95% Confidence interval

‡: Adjusted for annual average concentration of $PM_{10}$

Volume: 40, Article ID: e2018028

https://doi.org/10.4178/epih.e2018028

‡‡: Concentration of $NO_2$ in 1st (0–20th percentile): -10.46 ppb, 2nd (20th–40th percentile): 10.46-15.78 ppb, 3rd (40th –60th percentile): 15.78-19.76 ppb, 4th (60th –80th percentile): 19.76-29.87 ppb, 5th (80th –100th percentile): 29.87 ppb- in men. Concentration of $NO_2$ in 1st (0–20th percentile): -10.25 ppb, 2nd (20th–40th percentile): 10.25-15.06 ppb, 3rd (40th–60th percentile): 15.06-19.76 ppb, 4th (60th–80th percentile): 19.76-30.08 ppb, 5th (80th–100th percentile): 30.08 ppb- in women

#: Level of subjective stress. 1: 'I feel stress little', 2: 'I feel stress much', 3: 'I feel stress very much'

$PM_{10}$ 농도를 20 percentile 에 따라 5 분위로 나누어 독립변수로 두고 분석한 single-pollutant model 에서는 전반적으로 유의한 결과를 보이지 않았다. 30 세 이상 65 세 미만에서 percentile 이 증가할수록 스트레스 수준의 위험이 증가하는 경향을 보였지만, 가장 높은 5 분위의 남성에서만 통계적으로 유의한 결과를 보였다(Table 3). 분석 결과, '거의 느끼지 않는다'를 기준으로 '많이 느끼는 편이다'에 응답한 남성의 OR 은 1.57 (95% CI: 1.33-1.87), 여성의 OR 은 1.31 (95% CI: 1.11-1.54)였다.

**Table 3. Odds ratios (95% confidence interval) of subjective stress level in association with five quintiles for $PM_{10}$ stratified by sex, age in a single-pollutant model (continued)**

| Age group | $PM_{10}$ percentile‡ | level of stress# | Male OR† | Male 95% CI†† | Female OR† | Female 95% CI†† |
|---|---|---|---|---|---|---|
| < 30 | 1st | | ref | | | |
| | | 1 | 0.92 | (0.76–1.12) | 0.96 | (0.78–1.19) |
| | 2nd | 2 | 0.83 | (0.65–1.06) | 0.98 | (0.77–1.25) |
| | | 3 | 1.11 | (0.67–1.84) | 0.95 | (0.62–1.46) |
| | | 1 | 0.94 | (0.77–1.15) | 0.86 | (0.69–1.07) |
| | 3rd | 2 | 0.86 | (0.67–1.10) | 0.95 | (0.74–1.22) |
| | | 3 | 1.40 | (0.85–2.29) | 1.23 | (0.81–1.87) |
| | | 1 | 0.85 | (0.69–1.05) | 1.05 | (0.83–1.33) |
| | 4th | 2 | 0.82 | (0.63–1.06) | 1.08 | (0.82–1.40) |
| | | 3 | 1.03 | (0.61–1.73) | 1.20 | (0.77–1.87) |
| | 5th | 1 | 0.97 | (0.80–1.19) | 0.94 | (0.76–1.16) |

Volume: 40, Article ID: e2018028

https://doi.org/10.4178/epih.e2018028

| | | | | | | |
|---|---|---|---|---|---|---|
| | | **2** | 0.95 | (0.75–1.21) | 1.05 | (0.82–1.34) |
| | | **3** | 1.42 | (0.88–2.30) | 0.95 | (0.63–1.44) |
| **30-64** | **1st** | | | ref | | |
| | | **1** | 1.02 | (0.95–1.09) | 0.97 | (0.91–1.04) |
| | **2nd** | **2** | 1.04 | (0.95–1.13) | 1.01 | (0.93–1.09) |
| | | **3** | 1.14 | (0.96–1.35) | 1.05 | (0.89–1.23) |
| | | **1** | 0.96 | (0.89–1.04) | 1.00 | (0.93–1.08) |
| | **3rd** | **2** | 1.01 | (0.92–1.10) | 1.03 | (0.94–1.12) |
| | | **3** | 1.41 | (1.19–1.67) | 1.21 | (1.02–1.43) |
| | | **1** | 1.00 | (0.92–1.08) | 0.98 | (0.90–1.06) |
| | **4th** | **2** | 1.07 | (0.97–1.18) | 0.99 | (0.90–1.08) |
| | | **3** | 1.34 | (1.11–1.61) | 1.03 | (0.86–1.24) |
| | | **1** | 1.12 | (1.04–1.22) | 1.05 | (0.98–1.13) |
| | **5th** | **2** | 1.32 | (1.21–1.45) | 1.13 | (1.03–1.23) |
| | | **3** | 1.57 | (1.33–1.87) | 1.31 | (1.11–1.54) |
| **≥65** | **1st** | | | ref | | |
| | | **1** | 0.87 | (0.81–0.94) | 0.87 | (0.81–0.93) |
| | **2nd** | **2** | 0.86 | (0.77–0.96) | 0.80 | (0.73–0.87) |
| | | **3** | 0.78 | (0.59–1.02) | 0.83 | (0.70–0.99) |
| | | **1** | 0.87 | (0.79–0.96) | 0.82 | (0.75–0.89) |
| | **3rd** | **2** | 0.89 | (0.78–1.02) | 0.84 | (0.76–0.93) |
| | | **3** | 0.98 | (0.73–1.33) | 1.20 | (1.00–1.44) |
| | | **1** | 0.81 | (0.73–0.90) | 0.77 | (0.70–0.85) |
| | **4th** | **2** | 0.96 | (0.82–1.12) | 0.75 | (0.67–0.85) |
| | | **3** | 0.64 | (0.43–0.94) | 0.90 | (0.71–1.14) |

Volume: 40, Article ID: e2018028

https://doi.org/10.4178/epih.e2018028

| | | | | | |
|---|---|---|---|---|---|
| | **1** | 0.98 | (0.89–1.08) | 0.99 | (0.91–1.09) |
| **5th** | **2** | 1.00 | (0.87–1.15) | 0.94 | (0.84–1.05) |
| | **3** | 1.05 | (0.76–1.45) | 0.99 | (0.80 -1.24) |

CI, confidence interval; OR, odds ratio; $PM_{10}$, particulate matter <10 μm in size; ppb, parts per billion

† : Odds ratio (adjusted for temperature, humidity, income, age, marriage, cigarette smoking, educational level, economic activity, subjective health status, sleeping hours)

††: 95% Confidence interval

‡: Concentration of $PM_{10}$ in 0–20th percentile: 0–38.36 ㎍/㎥, 20th–40th percentile: 38.36–42.47 ㎍/㎥, 40th–60th percentile: 42.47–45.59 ㎍/㎥, 60th–80th percentile: 45.59–48.45 ㎍/㎥, 80th–100th percentile: 48.45㎍/㎥- in men. Concentration of $PM_{10}$ in 0–20th percentile: 0–38.36 ㎍/㎥, 20th–40th percentile: 38.36–42.44 ㎍/㎥, 40th–60th percentile: 42.44–45.47 ㎍/㎥, 60th–80th percentile: 45.47–48.45 ㎍/㎥, 80th–100th percentile: 48.45 ㎍/㎥- in women

#: Level of subjective stress. 1: ‘I feel stress little’, 2: ‘I feel stress much’, 3: ‘I feel stress very much

Volume: 40, Article ID: e2018028

https://doi.org/10.4178/epih.e2018028

**Discussion**

2013 년 지역사회건강조사 자료를 이용하여 조사 대상자의 253 개 보건행정 단위의 현재 거주지 주소를 바탕으로 대기오염 노출자료를 매칭하여 주관적 스트레스 정도를 분석하였다. 분석 결과, 사회경제적 활동이 활발한 남성과 여성 30 세 이상 65 세 미만의 연령에서 대기오염 노출 농도에 따른 분위가 증가할수록 스트레스 수준의 위험이 증가하였다.

지역사회 건강조사는 매년 고혈압, 당뇨병, 이상지질혈증, 뇌졸중, 심근경색증, 관절염 등 급성 및 만성질환에 대한 의사진단 여부를 조사한다. 질환의 유무는 일상 생활에서 느끼는 스트레스 수준과 삶의 질에도 큰 영향을 미친다[10]. 특히 관절염과 같은 활동에 제한이 있는 질환에 따라 느끼는 개인의 건강수준은 매우 낮을 것이다. 2013 년 조사 결과, 고혈압 의사진단을 받은 대상자는 총 55,632 명, 당뇨병은 20,922 명, 관절염은 31,675 명이었다. 이를 성별, 연령별로 나누어 분석하였을 때, 층화 변수에 따른 N 수가 매우 적어 통계적으로 유의한 결과를 내기 어렵다. 따라서, 질병 이환과 연관된 복합적인 지표로 주관적 건강수준을 ‘매우 좋음’, ‘좋음’, ‘보통’, ‘나쁨’, ‘매우 나쁨’ 로 응답한 변수를 이용하여 보정하였다.

현재까지 공중보건학적 관점에서 대기오염 물질에 대한 노출과 건강영향 연구는 주로 단일 대기오염물질에 중점을 두었다. 그러나 실제적으로 오염물질에 대한 노출은 다양한 배출원과 대기오염 물질에 노출되기 때문에 multi-pollutant model 접근법으로 나아갈 것을 권장하고 있다 [11]. 따라서 본 연구에서는 대기오염 물질 중 인체에 직접적인 영향을 미칠 수 있는 $NO_2$ 노출에 따른 single-pollutant model 과 $PM_{10}$ 을 보정한 multi-pollutant model 모두 분석하여 결과를 비교하였다. 남성과 여성 전체를 $NO_2$ 농도에 따라 5 분위로 나누어 분석하였을 때, 분위수가 증가할수록 주관적 스트레스 상태의 OR 이 증가하였고, 특히 여성보다 남성의 OR 이 더 높았다. 이러한 정신적 스트레스 정도는 평소 개인이 느끼는 건강관련 삶의 질과 연관이 있는데, 2005 년 일본에서 연구한 바에 따르면, 교통량이 많은 도로 근처에 거주하는 집단에서 건강관련 삶의 질이 낮았다고 보고하였다[12]. 이는 많은 교통량으로 인해 고농도의 $NO_2$ 에 노출되면 알레르기성비염[13], 천식 [14] 및 호흡기증상의 악화 [15] 등 단기노출로 인한 인체에 급성영향으로, 복합적으로 느끼게 되는 불쾌감 등의 주관적 건강상태와 건강 관련 삶의 질에도 영향을 미칠 수 있다는 것을 시사한다. 실제로 인체에 흡입된 대기오염물질은 직접적으로 지질과 단백질의 산화촉진제로 또는 자유라디칼(free radical)로 작용하여 산화 스트레스를 촉진시키고 염증반응을 유도하게 된다 [16]. 인체 내 자유라디칼 농도가 증가하면 산화 손상

Volume: 40, Article ID: e2018028

https://doi.org/10.4178/epih.e2018028

스트레스로 인해 죽상 동맥 경화증, 심장 마비, 만성 염증성 질환과 파킨슨병, 알츠하이머와 같은 중추신경계 장애를 유발할 수 있다[17]. 이러한 신체 내 손상은 개인이 일상생활에서 느끼는 신체기능의 저하로 인해 정신적으로 영향을 미칠 수 있다.

남성과 여성에서 사회 인구학적 연령에 따른 결과를 보기 위해 남성과 여성을 30 세 미만, 30 세 이상 65 세 미만, 65 세 이상으로 나누어 분석하였다. 30 세 이상 65 세 미만의 남성과 여성은 사회 및 경제 활동이 가장 활발하고, 30 세 미만의 연령에 비해 상대적으로 신체적 기능의 감퇴가 시작되는 시기이다. 모든 연령군에서 $NO_2$ 농도의 분위수가 증가할수록 스트레스 정도의 OR 이 증가하였다. 특히, multi-pollutant model 에서 30 세 이상 65 세 미만의 남성에서 OR 이 뚜렷하게 증가하였고, 모두 통계적으로 유의한 결과를 보였다. 이 시기는 활동성이 가장 많고, 사회 및 경제활동이 가장 활발한 시기로써 일상생활에서 실내· 외 대기오염에 노출되기 쉽다. 또, 사회 경제적 수준에 따라 상대적으로 다른 연령군에 비해 민감하게 반응하여 30 세 미만 연령군과 65 세 이상 연령군에 비해 민감집단이기 때문일 것이라 판단된다. 질병 이환에 따른 복합적인 지표로 주관적 건강상태에 따른 스트레스 정도를 분석한 결과, 건강상태가 나빠질수록 OR 이 뚜렷하게 증가하였다. 하루 평균 수면시간에 따른 스트레스 정도를 분석한 결과, 남성과 여성모두 30 세 이상 65 세 미만의 연령에서 하루 평균 수면시간이 감소할수록 OR 이 뚜렷하게 증가하였다.

본 연구에서는 $PM_{10}$ 의 영향을 보기 위해 농도에 따라 5 분위로 나누어 분석을 시행하였다. Single-pollutant model 결과, 30 세 이상 65 세 미만의 남성에서 $PM_{10}$ 의 분위수가 높아질수록 스트레스 정도의 OR 이 뚜렷하게 증가하였으나 제 4 분위와 5 분위에서만 통계적으로 유의한 결과를 보였다. 여성에서도 분위수가 높아질수록 OR 이 증가하는 경향을 보였으나 가장 높은 5 분위에서만 통계적으로 유의한 결과를 보였다(Table 3). 이는 사람에게 $PM_{10}$ 보다 $NO_2$ 의 영향이 크다는 것을 보여준다. 도로 위 자동차의 배출가스는 국내 대기오염의 주 원인 중 하나이다. 도로의 교통량과 대기오염 물질의 상관성을 조사한 결과, $PM_{10}$ 보다 $NO_2$ 에서 상관성이 매우 높게 나왔고, 이는 $PM_{10}$ 농도는 자동차 배출가스의 영향을 직접적으로 반영하지 않는다는 결과를 보여준다[18]. 우리는 일상 생활에서 각종 교통수단을 자주 이용할 뿐만 아니라 수많은 교통량에서 배출되는 가스에 직ㆍ간접적으로 노출되고 있다. 따라서, $NO_2$ 노출로 인한 신체적 및 정신적 피해를 감소시키기 위해서 교통수단과 다양한 대기오염 물질의 배출원에 대한 규제가 필요하다는 것을 시사한다.

Volume: 40, Article ID: e2018028

https://doi.org/10.4178/epih.e2018028

본 연구는 지역사회 건강조사에서 시행되는 통계자료가 253 개 보건행정 지역단위로 산출된다는 장점을 이용하여 253 개 지역의 대기오염 농도자료를 결합하여 분석하였다는 강점이 있다. 그러나 현재 일반 연구자들에게 제공되는 지역사회 건강조사 통계자료는 조사가 시행된 날짜 변수가 제공되지 않기 때문에 우리는 연평균 대기오염농도를 산출하여 상대적으로 long-term exposure 에 해당하는 결과값을 얻었다. 대기오염 물질 단기노출은 인체 내 산화 스트레스 손상을 일으키고 [19], 고농도에 노출되게 되면 급성 스트레스에 반응 시 분비되는 호르몬인 cortisol, norepinephrine 농도가 증가한다고 보고하였다 [20]. 따라서, 지역사회 건강조사 대상자의 조사 시행 날짜에 따른 대기오염 단기노출로 인한 건강영향을 보기 위해서 일별자료를 결합하여 분석한다면 더 큰 영향을 볼 수 있을 것이라고 판단된다. 본 연구자는 현재 가용한 대기오염 농도자료가 2013 년으로 제한되어 2013 년의 지역사회 건강조사 통계자료만 이용하였지만, 추후 다른 연도의 대기오염 농도자료가 구축되면 연도별 농도변화에 따른 건강영향을 분석할 수 있을 것이다. 또, 본 연구에서 대상자들의 대기오염 노출은 실외 대기오염노출에 근거한 자료이다. 노출에 많은 영향을 미치는 가정과 직장 내 환경에서의 노출을 반영하지 못했다는 제한점이 있다. 매년 질병관리본부에서 주관하는 지역사회건강조사는 전국적으로 8 월 말에 시작하여 10 월 말에 종료하여 실질적으로 연중 두, 세 달 동안만 시행된다. 따라서, 253 개 지역의 연평균 $NO_2$ 농도를 결합하여 분석하였지만, 연중 계절성을 고려하지 못하였다는 제한점이 있다.

국내의 대기오염에 의한 건강영향 연구 다수는 국내 자동 측정망에서 수집된 대기환경자료를 이용하였다. 국내 자동 측정망에서 수집된 대기물질 농도자료는 측정점의 밀도가 낮은 지역의 농도는 정확성이 낮고, 자료가 미비하다는 한계점을 가지고 있다. 따라서 본 연구에서 이용한 KAQFS 자료를 이용하여 지역사회 건강조사의 보건행정 지역단위로 대기오염 물질 농도를 산출하였고, 시・군・구 단위의 해상도로 대기오염 노출에 의한 스트레스 정도를 분석하여 기존의 연구에서의 한계점을 보완하였다. 그러나 본 연구에서 사용한 대기오염 노출값은 실제 측정자료가 아닌 CMAQ 모델값을 이용하였다는 제한점이 있다. 모델링 과정에서 입력값인 국내 배출량 자료는 2008 년 국립환경과학원에서 제공하는 자료를 이용하였지만, 전국 226 개 측정자료만을 사용하였고, 전국의 배출원으로부터 오염물질의 배출량을 반영하는 데 한계가 있다.

기존의 국내 연구들은 대기오염으로 인한 건강 관련 질병발생과 이환 지표에 집중하여 보고되었지만, 본 연구에서는 질병 발생수준 이전에 일상 생활에서 느끼는 정신적

감정상태에 대한 영향을 분석하였다는 데 의의가 있다. 본 연구는 가용할 수 있는 단년도 모델링 자료와 지역사회건강조사를 결합하여 대기오염 노출에 대한 주관적 스트레스 영향을 연구하였다. 추후 보건행정단위의 가용한 대기오염 노출자료를 확대하여 지역단위의 건강지표를 이용하여 연구한다면, 매년 보건행정 지역단위에서 대기오염 노출로 인한 개인 및 지역사회의 건강관련 주관적 삶의 질의 수준을 평가하여 보건 및 복지정책 수립 시 유용한 근거로 이용될 수 있을 것이다.

## Conclusions

본 연구에서는 30 세 이상 65 세 미만의 남성과 여성에서 $NO_2$ 농도와 주관적 스트레스 정도가 관련이 있었다. 2013 년 환경부에서 공개한 16 개 시도의 연평균 $NO_2$ 측정 결과, 가장 높았던 서울지역이 35 ppb, 가장 낮았던 제주지역은 8 ppb 로 지역 간의 차이가 컸다. 이는 집단의 수준에서 사회 경제적 요인에 따라 질병 발생 및 이환에 영향을 미칠 수 있는 정신적 감정상태의 차이도 크다는 것을 시사한다. 우리는 매년 보건행정 지역단위로 산출되는 지역사회 건강조사 통계자료를 이용하여 지역 간 대기오염 농도에 따른 보건정책 수립 시, 과학적 근거에 기반을 둔 좋은 정책을 만들 수 있다고 생각한다.

## Acknowledgements

**This work was supported by the Korea Centers for Disease Control and Prevention (KCDC).**

## Conflict of interest

The author has no conflicts of interest to declare for this study.

Volume: 40, Article ID: e2018028

https://doi.org/10.4178/epih.e2018028

**Reference**

[1] Brook RD, Franklin B, Cascio W, Hong Y, Howard G, Lipsett M, et al. Air pollution and cardiovascular disease: a statement for healthcare professionals from the Expert Panel on Population and Prevention Science of the American Heart Association. Circulation 2004;109(21):2655-2671.

[2] Lim YH, Kim H, Kim JH, Bae S, Park HY, Hong YC. Air pollution and symptoms of depression in elderly adults. Environ Health Perspect 2012;120(7):1023-1028.

[3] Jung CR, Lin YT, Hwang BF. Air pollution and newly diagnostic autism spectrum disorders: a population-based cohort study in Taiwan. PLoS One 2013;8(9):e75510.

[4] Ritz B, Lee PC, Hansen J, Lassen CF, Ketzel M, Sorensen M, et al. Traffic-related air pollution and Parkinson's disease in Denmark: a case-control study. Environ Health Perspect 2016;124(3):351-356.

[5] Chang KH, Chang MY, Muo CH, Wu TN, Chen CY, Kao CH. Increased risk of dementia in patients exposed to nitrogen dioxide and carbon monoxide: a population-based retrospective cohort study. PLoS One 2014;9(8):e103078.

[6] World Health Organization. Ambient air pollution: a global assessment of exposure and burden of disease. 2016. Available at apps.who.int/iris/bitstream/10665/250141/1/9789241511353-eng.pdf (Accessed on Apr 27 2018).

[7] Oglesby L, Künzli N, Monn C, Schindler C, Ackermann-Liebrich U, Leuenberger P, SAPALDIA Team. Validity of annoyance scores for estimation of long term air pollution exposure in epidemiologic studies: the Swiss Study on Air Pollution and Lung Diseases in Adults (SAPALDIA). Am J Epidemiol 2000;152(1):75-83.

[8] Kim YT, Choi BY, Lee KO, Kim H, Chun JH, Kim SY, et al. Overview of Korean Community Health Survey. J Korean Med Assoc 2012;55(1):74-83.

[9] Kwon GY, Lim DS, Park EJ, Jung JS, Kang KW, Kim YA, et al. Assessment of applicability of standardized rates for health state comparison among areas: 2008 community health survey. J Prev Med Public Health 2010;43(2):174-184.

[10] Saarni SI, Härkänen T, Sintonen H, Suvisaari J, Koskinen S, Aromaa A, et al. The impact of 29 chronic conditions on health-related quality of life: a general population survey in Finland using 15D and EQ-5D. Qual Life Res 2006;15(8):1403-1414.

[11] Dominici F, Peng RD, Barr CD, Bell ML. Protecting human health from air pollution: shifting from a single-pollutant to a multipollutant approach. Epidemiology 2010;21(2): 187-194.

Volume: 40, Article ID: e2018028

https://doi.org/10.4178/epih.e2018028

[12] Yamazaki S, Sokejima S, Nitta H, Nakayama T, Fukuhara S. Living close to automobile traffic and quality of life in Japan: a population-based survey. Int J Environ Health Res 2005;15(1):1-9.

[13] Weiland SK, Mundt KA, Rückmann A, Keil U. Self-reported wheezing and allergic rhinitis in children and traffic density on street of residence. Ann Epidemiol 1994;4(3):243-247.

[14] Duhme H, Weiland SK, Keil U, Kraemer B, Schmid M, Stender M, et al. The association between self-reported symptoms of asthma and allergic rhinitis and self-reported traffic density on street of residence in adolescents. Epidemiology 1996;7(6):578-582.

[15] Ciccone G, Forastiere F, Agabiti N, Biggeri A, Bisanti L, Chellini E, et al. Road traffic and adverse respiratory effects in children. SIDRIA Collaborative Group. Occup Environ Med 1998;55(11):771-778.

[16] Rahman I, MacNee W. Oxidative stress and regulation of glutathione in lung inflammation. Eur Respir J 2000;16:534-554.

[17] Kampa M, Castanas E. Human health effects of air pollution. Environ Pollut 2008;151(2):362-367.

[18] Lau J, Hung WT, Cheung CS. Interpretation of air quality in relation to monitoring station's surroundings. Atmospheric Environment 2009;43(4):769-777.

[19] Li W, Wilker EH, Dorans KS, Rice MB, Schwartz J, Coull BA, et al. Short- Term Exposure to Air Pollution and Biomarkers of Oxidative Stress: The Framingham Heart Study. J Am Heart Assoc 2016;5(5):e002742.

[20] Li H, Cai J, Chen R, Zhao Z, Ying Z, Wang L, et al. Particulate matter exposure and stress hormone levels: a randomized, double-blind, crossover trial of air purification. Circulation 2017;136(7):618-627.
